# Supplementary material for: Evidence of human influence on Northern Hemisphere snow loss
Source: Nature. 2024 Jan 10;625(7994):293–300. doi: 10.1038/s41586-023-06794-y (PMC10781623; doi:10.1038/s41586-023-06794-y)
Supplement: Supplementary file 1 — Supplementary Information [file 41586_2023_6794_MOESM1_ESM.pdf]

nature portfolio

https://doi.org/10.1038/s41586-023-06794-y

# Supplementary information

# Evidence of human influence on Northern Hemisphere snow loss

In the format provided by the
authors and unedited

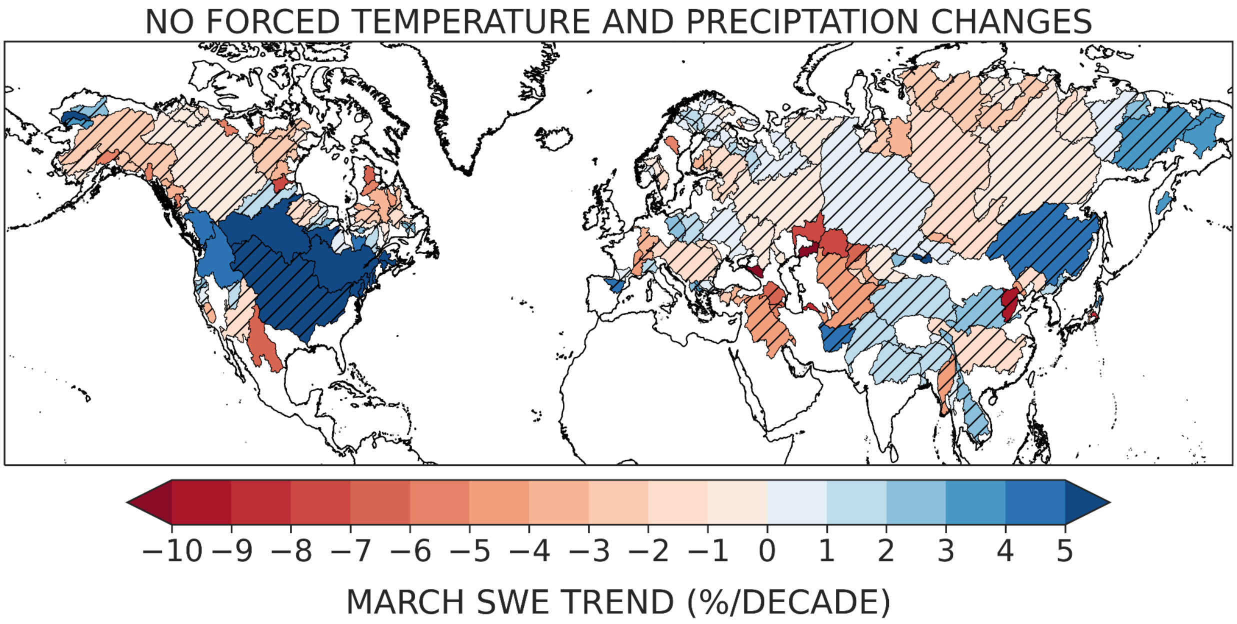


**SI Fig. 1 | Few significant long-term snowpack trends absent human-caused climate change.** Reconstructed ensemble mean trend in March SWE from 1981 to 2020 with forced changes to temperature and precipitation removed (see Methods). Hatching indicates basins where fewer than 80 percent of estimates agree on the direction of the trend. Maps were generated using cartopy v0.18.0. River basin boundaries come from the Global Runoff Data Centre's Major River Basins of the World database.[44]

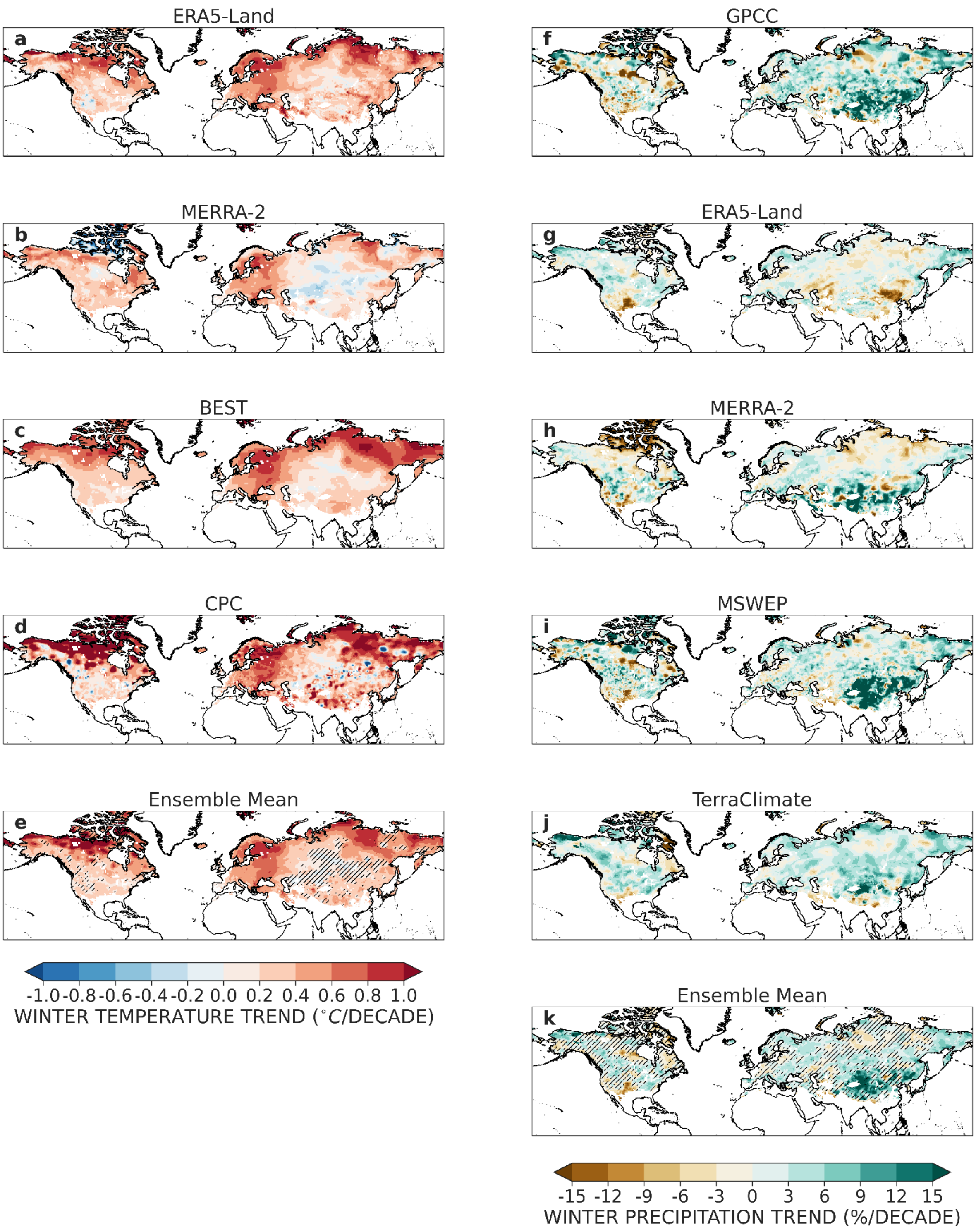


**SI Fig. 2 | Observed trends in winter temperature and precipitation.** Trends in November-March average temperature (**a-e)** and total precipitation (**f-k**) from 1981 to 2020 in the gridded products used in the SWE reconstructions. Hatching in the ensemble mean subplots indicates grid cells where at least 1 (2) products(s) disagrees on the sign of the temperature (precipitation) trend. Maps were generated using cartopy v0.18.0.

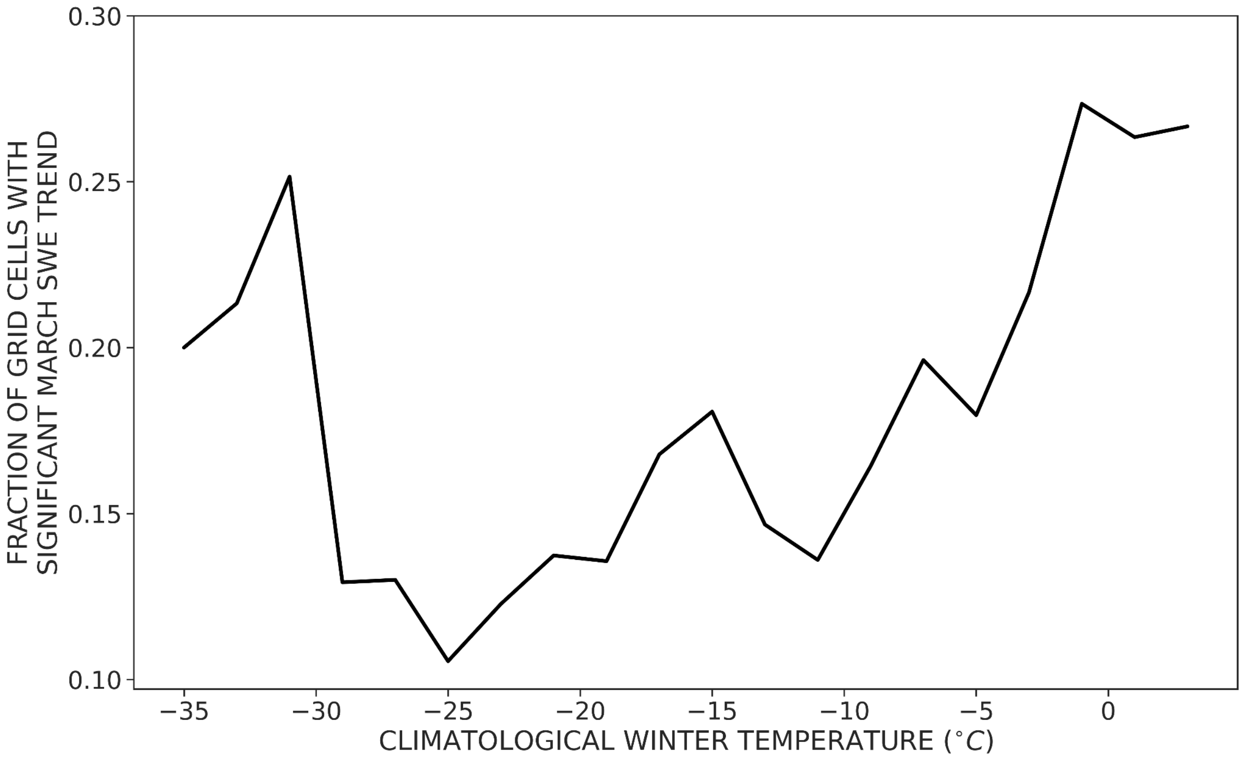


**SI Fig. 3 | Most coherent long-term SWE trends observed where climatological winter temperatures are near the freezing point.** Fraction of all product-grid cells that have shown a statistically significant (Mann-Kendall $p<0.05$) trend in March SWE from 1981-2020.

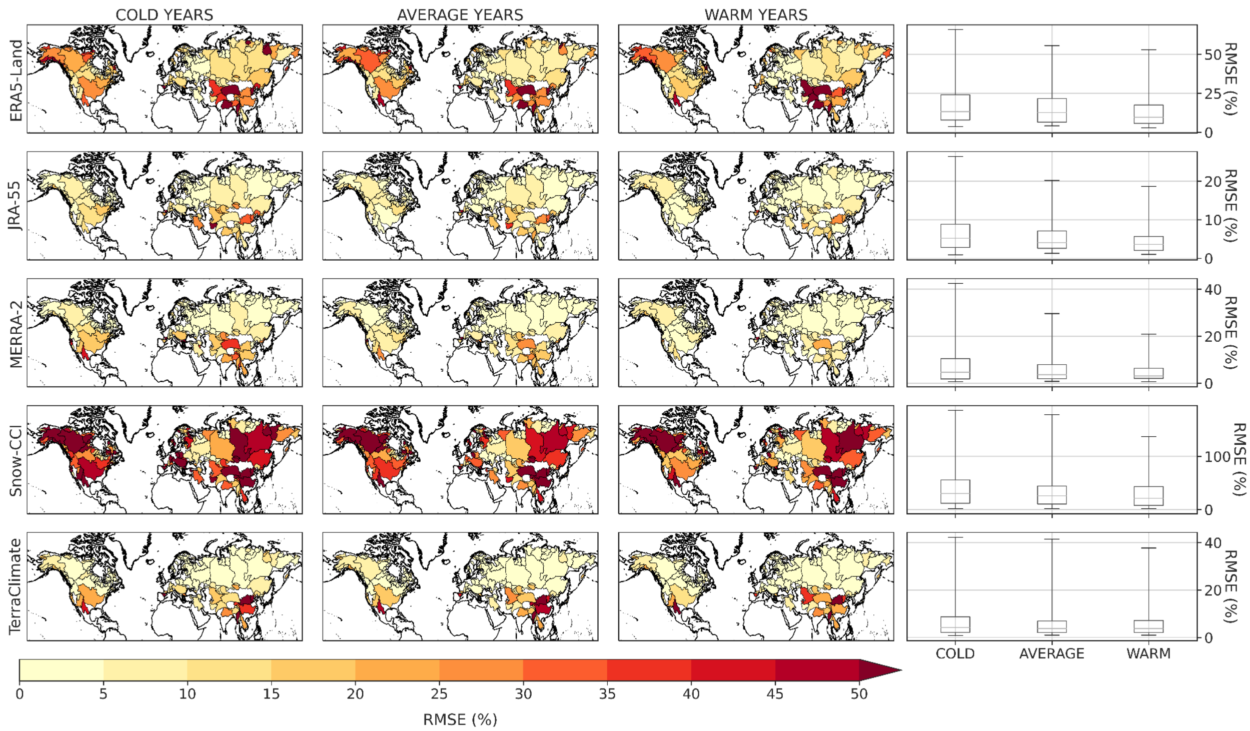


**SI Fig. 4 | Reconstruction skill is comparable across a gradient of winter temperatures.** RMSE in the 10 coldest, 10 warmest, and 20 "average" years in each basins from 1981-2020. Boxplots show the distribution of skill across basins in each temperature category, with the line indicating the median basin skill, the box the interquartile range, and the whiskers the 2.5th and 97.5th percentiles. Maps were generated using cartopy v0.18.0. River basin boundaries come from the Global Runoff Data Centre's Major River Basins of the World database.[44]

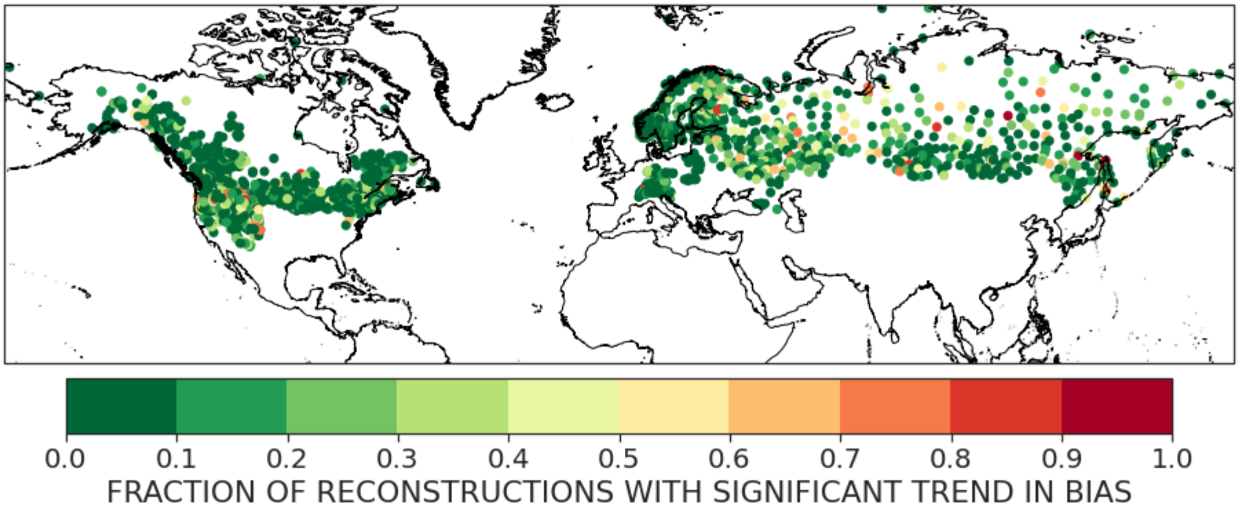


**SI Fig. 5 | Minimal bias in reconstructed trends for out-of-sample *in situ data*.** Fraction of empirical reconstructions of *in situ* March SWE with statistically significant (Mann-Kendall $p<0.05$) trends in bias relative to *in situ* observations. Maps were generated using cartopy v0.18.0.

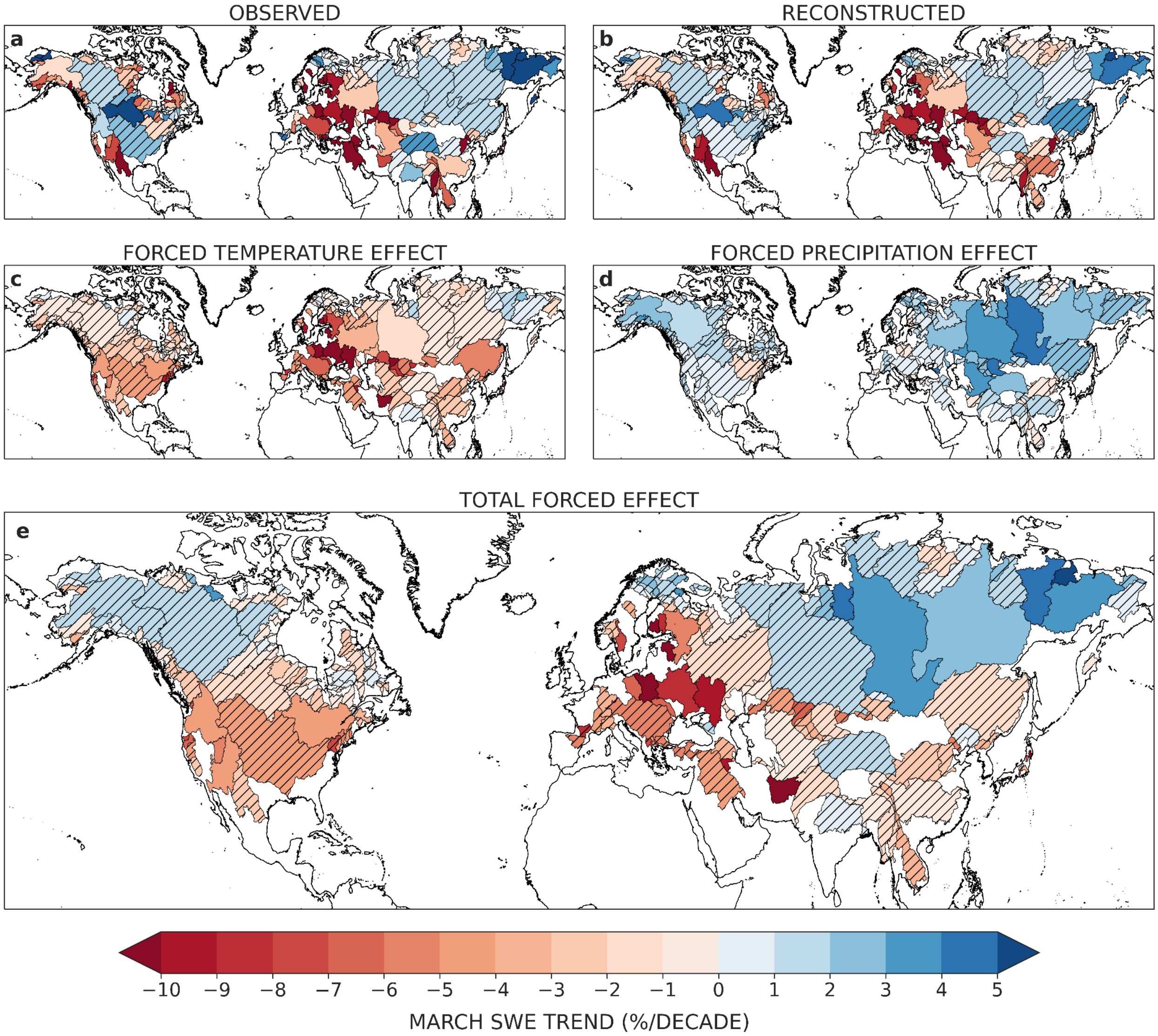


**SI Fig. 6 | Estimated effects of human-forced temperature and precipitation trends on basin-scale snow changes similar when using only one realization of each climate model. a**, Average observed 1981-2020 March SWE trends from 5 long-term SWE data products in 169 major Northern Hemisphere river basins. **b**, As in (a), but for our observation-based reconstructions. **c**, Effect of anthropogenically-forced T changes on March SWE trends, given by the ensemble mean difference between the statistically-reconstructed historical trend and the reconstructed trend with forced changes to T removed using the first realization of each climate model. **d**, As in (**c**), but for forced P changes. **e**, As in (c) and (d) but for forced changes to both T and P. Hatching indicates basins where fewer than 80% of observations or reconstructed estimates agree on the sign of the trend or forced effect. Maps were generated using cartopy v0.18.0. River basin boundaries come from the Global Runoff Data Centre's Major River Basins of the World database.[44]

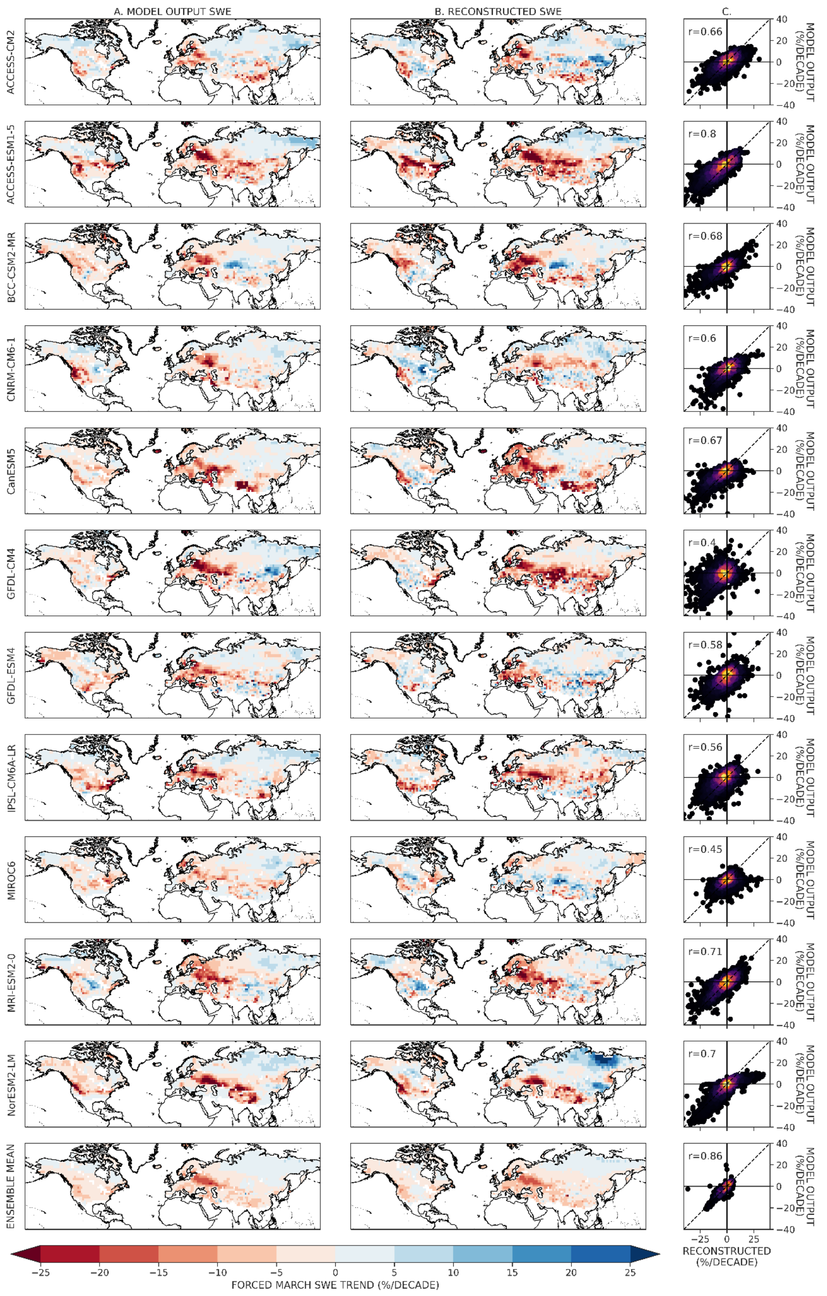


**SI Fig. 7 | The Random Forest snowpack reconstruction methodology exhibits high skill based on a perfect model framework in individual models and the ensemble mean.** Forced (HIST minus HIST-NAT) trends in March SWE from 1981-2020 based on (**a**) climate model SWE output and (**b**) counterfactual SWE estimated using HIST-NAT temperature and precipitation and Random Forest model. **c**, Scatterplot of reconstructed versus original trends, where each dot represents a grid cell. Points are colored by their density. Dashed line denotes perfect agreement between reconstructed and original trends. Pearson's correlation is shown in upper left corner. Maps were generated using cartopy v0.18.0.
